# Hierarchical Assembly of Multifunctional Oxide-based Composite Nanostructures for Energy and Environmental Applications

**DOI:** 10.3390/ijms13067393

**Published:** 2012-06-15

**Authors:** Pu-Xian Gao, Paresh Shimpi, Haiyong Gao, Caihong Liu, Yanbing Guo, Wenjie Cai, Kuo-Ting Liao, Gregory Wrobel, Zhonghua Zhang, Zheng Ren, Hui-Jan Lin

**Affiliations:** Nanomaterials Science Laboratory, Department of Chemical, Materials and Biomolecular Engineering & Institute of Materials Science, University of Connecticut, Storrs, CT 06269, USA; E-Mails: paresh.shimpi@gmail.com (P.S.); haiyonggao@gmail.com (H.G.); caihong.liu@engr.uconn.edu (C.L.); yanbing.guo@ims.uconn.edu (Y.G.); andy.cai@gradmail.ims.uconn.edu (W.C.); kuo-ting.liao@uconn.edu (K.-T.L.); gregory.wrobel@uconn.edu (G.W.); zhonghua.zhang@ims.uconn.edu (Z.Z.); zheng.ren@uconn.edu (Z.R.); hui-jan.lin@uconn.edu (H.-J.L.)

**Keywords:** nanocomposite, nanowire/particle/film, self-assembly, metal oxide, band-gap engineering, catalysis, chemical sensors, thermal engineering

## Abstract

Composite nanoarchitectures represent a class of nanostructured entities that integrates various dissimilar nanoscale building blocks including nanoparticles, nanowires, and nanofilms toward realizing multifunctional characteristics. A broad array of composite nanoarchitectures can be designed and fabricated, involving generic materials such as metal, ceramics, and polymers in nanoscale form. In this review, we will highlight the latest progress on composite nanostructures in our research group, particularly on various metal oxides including binary semiconductors, ABO_3_-type perovskites, A_2_BO_4_ spinels and quaternary dielectric hydroxyl metal oxides (AB(OH)_6_) with diverse application potential. Through a generic template strategy in conjunction with various synthetic approaches— such as hydrothermal decomposition, colloidal deposition, physical sputtering, thermal decomposition and thermal oxidation, semiconductor oxide alloy nanowires, metal oxide/perovskite (spinel) composite nanowires, stannate based nanocompostes, as well as semiconductor heterojunction—arrays and networks have been self-assembled in large scale and are being developed as promising classes of composite nanoarchitectures, which may open a new array of advanced nanotechnologies in solid state lighting, solar absorption, photocatalysis and battery, auto-emission control, and chemical sensing.

## 1. Introduction

The search for strategies to rationally and scalably assemble complex functional oxide-based nanostructures in hierarchical fashion have attracted significant research interests and efforts due to the request in-demand and their potential applications in advanced nanodevices and nanosystems toward energy, environmental and sensing sectors. Hierarchical nanostructures can be classified according to the dimensions of nano-building blocks and the assembled hierarchical structures [1,2]. A number of novel hierarchical nanostructures have been designed and synthetically created [3–5]. For instance, one dimensional (1D) nanoscale building blocks, such as nanowires, nanorods, and nanotubes, can be self-assembled into hierarchical nanostructures in the form of axial, radial, and branched nanostructures [1,3]. Recently, monodisperse colloidal nanocrystals in solution environment were discovered to be able to self-organize into three-dimensional (3D) superstructures by virtue of nanocrystal interlocking chain formation and followed by chain self-assembly as encoded in the nanocrystals’ octapod shape [5]. With the incorporation of zero dimensional (0D) nanoparticles and two dimensional (2D) nanofilms, an even richer family of hierarchical nanostructures forms and holds a great potential for future utilization in nanotechnology. On the other hand, dissimilar nanomaterials, when integrated together, will enable the integration of multiple important functions and allow exhibition of more complex and fascinating novel properties as the foundation for new devices and systems. A famous example could go to the discovery of giant magnetoresistance multilayer nanofilm “composite” [6,7], which has signified the new era of information storage technology. In light of these discoveries, we have focused on designing and engineering a new class of hierarchical oxide-based composite nanostructures for enhancing scientific understanding and expediting technology progress in ultraviolet (UV) lighting, solar absorption and utilization, emission control and chemical sensing.

Illustrated in Schematic 1 is the hierarchical assembly strategy of composite nanostructures used in the work reviewed here. First, a generic template strategy is utilized throughout, with templates being either nanostructure or microstructure building blocks, which will be grown first. Second, the nano- or microstructured templates are processed with additional combination and integration of various synthetic approaches such as hydrothermal decomposition, colloidal deposition, physical sputtering, thermal decomposition and thermal oxidation. Examples of success are reviewed below including three classes of oxide-based composite nanostructures: solution-processed semiconductor alloy nanowire arrays [8,9], metal oxide/perovskite (or spinel) nanowire arrays [10–14], and metal hydroxystannate and their derivative stannate composite nanofilms [15–17]. Furthermore, through a simple thermal oxidation over metal coated three-dimensional templates such as ZnO-Cu nanowire arrays and two-dimensional ones like surface heterogeneous metallurgical pattern on Cu-Zn alloy, arrays or networks of ZnO-CuO nanoscale heterojunction have been enabled in large scale [18,19]. Photoluminescence, photocatalysis, catalytic CO oxidation and chemical sensing properties have been investigated, demonstrating promising new applications.

## 2. Self-Assembly and Utilization of Oxide-based Nanoarchitectures

### 2.1. Single-Component Template Nanostructures

**Figure 1:**
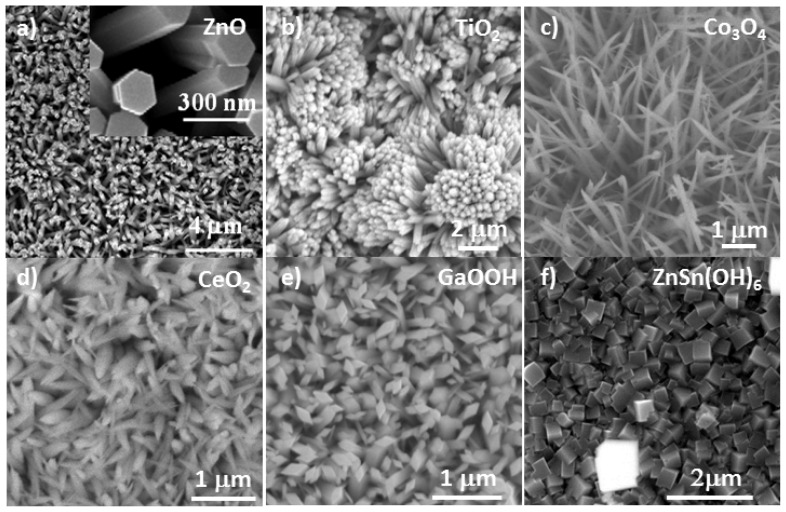
A set of SEM images displaying the single-component nanostructure templates made by solution phase process (**a**) ZnO nanowire arrays; (**b**) TiO_2_ nanowire arrays; (**c**) Co_3_O_4_ nanowire arrays; (**d**) CeO_2_ nanorod arrays; (**e**) GaOOH nanorod arrays; (**f**) ZnSn(OH)_6_ nanocubes.

Focusing on metal oxide and semiconductor nanostructures, a series of techniques have been developed to fabricate various functional oxide based homogeneous (single-component) template nanostructures. The techniques range from solution phase approach to vapor phase approach. It is well known that solution process is a low cost and easy-scale-up method for fabricating nanostructures. As a result, the solution phase processes have been successfully used for growing nanostructures made from a diverse array of functional oxides, such as ZnO, CeO_2_, TiO_2_, Co_3_O_4_, GaOOH, and ZnSn(OH)_6_ [20–23]. Figure 1 shows a set of nanostructure templates grown using the solution phase approach.

**Figure 2:**
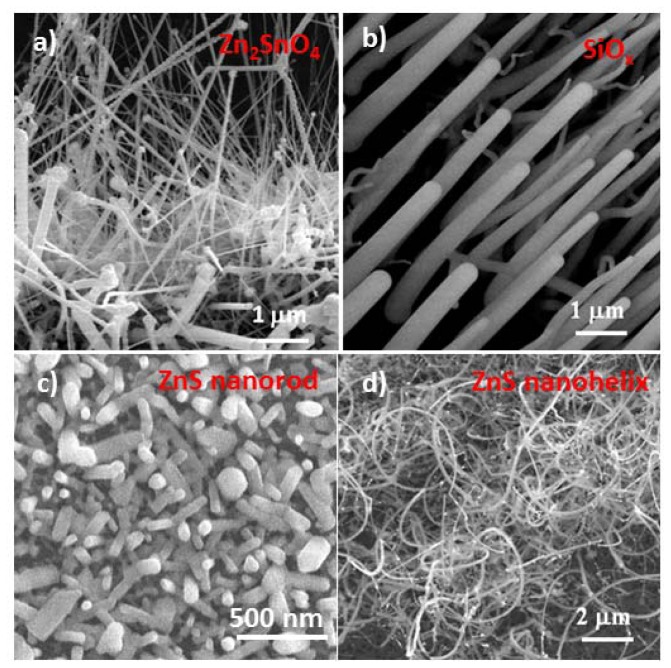
A set of SEM images displaying the nanostructure templates fabricated by vapor phase process (**a**) Zn_2_SnO_4_ nanowire arrays, (**b**) SiO*_x_* nanowire arrays, (**c**) ZnS nanorods, and (**d**) ZnS helical nanostructures.

On the other hand, vapor deposition has also been used for the fabrication of oxides and semiconductors such as ZnO, SiO_2_, Ga_2_O_3_, SnO_2_, ZnS, CdS, CdSe, and Zn_2_SnO_4_ [24–29]. This is an ideal method for achieving a better crystal quality than solution approach, but mostly applies in the binary system due to the difficulty in maintaining phase composition and stoichiometry of the material at vapor phase, with the exception of the laser ablation approach. It is worth pointing out that, as discussed in various literature, through control of flow dynamics and carrier gas concentration, in addition to temperature parameter, different morphological and oriented nanostructures from the same compounds could be achieved [28,30]. Figure 2 displays examples of Zn_2_SnO_4_, SiO_2_, and ZnS nanostructures. Through flowing with reducing atmosphere such as CO under low pressure, it is found that the [001] oriented ZnS nanofilm could be converted into dispersive ZnS nanorods (Figure 2c) [31], similar to the ZnO nanofilm-nanowire conversion case [30]. It has been found that, through tuning the CO reducing carrier gas, the surface energy state sequence in the three basic crystal facets (*i.e.*, {0001}, {01–10}, {11–20}) in ZnO could be changed, leading to the growth habit change. The growth of [0001] oriented ZnO nanowires with no CO involvement has been tuned to the populated formation of [01–10] oriented zig-zag nanobelts in the presence of CO carrier gas, in addition to the N_2_, the normal carrier gas. On the other hand, by changing the laminar flow to turbulent flow, ZnS nanostructures could be tuned from straight nanowires into helical nanostructures (Figure 2d), which may be due the flow dynamics induced concentration and temperature gradient variations [28].

**Figure 3:**
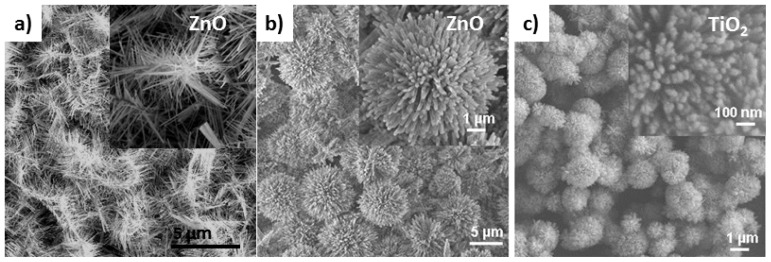
Single-component nanostructures in hierarchical fashion: (**a**) ZnO nanodendrite arrays; (**b**) ZnO koosh balls; and (**c**) TiO_2_ koosh balls.

Nevertheless, both solution and vapor deposition techniques have been utilized as vital methods for enabling various functional oxide nanostructures in hierarchical fashion, such as nanowire arrays in Figures 1,2, dendrite nanostructures [32,33], and koosh ball nanostructures [34], as displayed in Figure 3.

### 2.2. Two or Multi-Component Composite Nanostructure Assembly

With the addition of foreign elements/compounds into (or onto) pristine homogeneous nanostructures by either solid solution (doping or alloying) or interfacing/layering, potential improvement or multiplication of functional characteristics in single-component materials could be achieved. As mentioned earlier, a significant case was the discovery of giant magnetoresistance effect by periodically sandwiching two dissimilar nanofilms together, forming multilayer composite thin film. In our group, we have a major research focus on the design, fabrication and utilization of heterogeneous composite nanostructures. Here we would like to highlight three specific examples: semiconductor oxide alloy nanowires, semiconductor/perovskite (spinel) composite nanowires, and stannate based composite nanofilms, which have abundant important implications toward energy, environmental and sensing applications.

#### 2.2.1. Solution-Processed Mg Alloying of ZnO Nanorod Arrays

**Figure 4:**
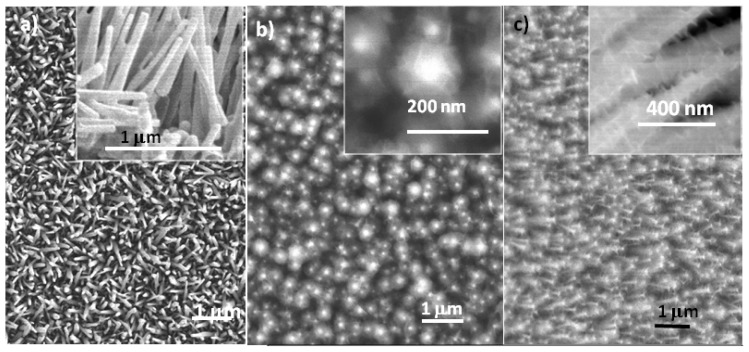

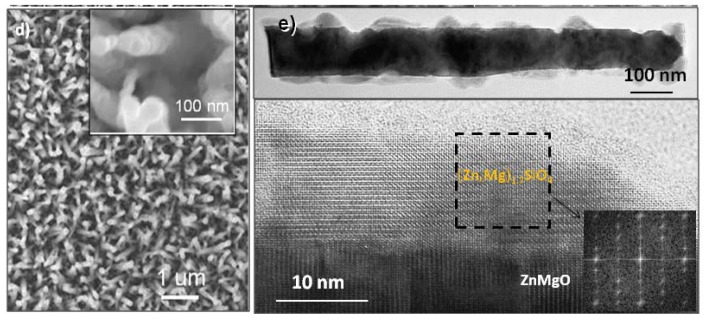
Solution processed ZnMgO nanorods using sequential hydrothermal synthesis: (**a**) top view of as-prepared ZnO nanorods, inset is the side view of ZnO nanowires; (**b**) top view of ZnMgO nanorods after 2-step hydrothermal synthesis; and (**c**) side view of ZnMgO nanorods; (**d**) ZnMgO nanorods after a 900 °C ambient annealing for 5 min; and (**e**) TEM images of a single ZnMgO nanorods surrounded by an epitaxial layer of (Zn,Mg)_1.7_SiO_4_.

Shown in Figure 4 is a set of electron micrographs displaying the solution-processed ZnMgO nanorods using a 2-step sequential hydrothermal process [9]. Figure 4a is a top view SEM image of as-prepared ZnO nanorod arrays and inset is the side view of ZnO nanorods, which clearly identifies the closely packed nature and dimensions (80–100 nm wide, 1–1.5 μm long) of nanowire arrays. After a second-step sequential hydrothermal synthesis based on ZnO nanorod arrays, ZnMgO nanorods were successfully achieved with dendritic surface branches as suggested from the top and side view SEM images in Figure 4b,c, respectively. The dendrite branches were revealed to be amorphous MgO by high resolution transmission electron microscopy (HRTEM) results and the core has been successfully alloyed with Mg, which was confirmed in X-ray diffraction (XRD), X-ray photoelectron spectroscopy (XPS) and photoluminescence (PL) spectroscopy, which are further described in Figure 4.

To increase the Mg alloying content in ZnO nanorod arrays, a 900 °C ambient annealing treatment has been conducted [8]. Figure 4d shows the ZnMgO nanorods after a 5-min 900 °C ambient annealing. The TEM results in Figure 4e clearly identified the formation of single crystal like core-shell nanowire: the ZnMgO nanorod was surrounded by an epitaxial layer of (Zn,Mg)_1.7_SiO_4_, which was identified in the high resolution TEM image and the FFT selected area electron diffraction pattern.

**Figure 5:**
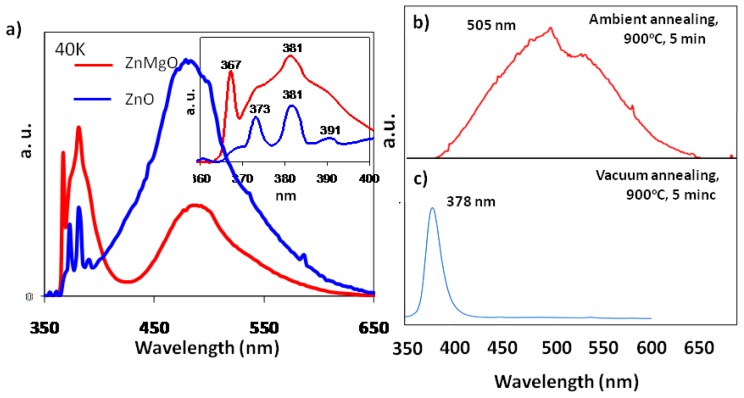
A set of photoluminescence spectra corresponding to: (**a**) as-prepared solution processed ZnMgO nanowire arrays measured at 40 K; (**b**) 900 °C ambient annealed ZnMgO nanowires measured at room temperature; (**c**) 900 °C vacuum (1 × 10^−2^ torr.) annealed ZnMgO nanowires measured at room temperature.

Both room temperature (298 K) and low temperature (40 K) photoluminescence results (Figure 5a) revealed an enhanced and blue-shifted near-band-edge (NBE) UV emission for the MgO/ZnMgO nanowires compared to those of the pure ZnO nanowire arrays, which proves the success of solution phase alloying in ZnO nanowire arrays after the 2nd step 155 °C deposition of MgO [9]. This enhancement might be due to the 155 °C high-pressure hydrothermal process and the amorphous MgO layer in the MgO/ZnO nanowires. For Mg ions to replace Zn ions as a route of substitution diffusion, it is clearly not energetically favorable at temperatures as low as 155 °C; however the PL data together with the XRD results confirmed the possible alloying success at low temperature given that a specific template of densely-packed ZnO nanowire arrays was used during the low temperature alloying process. Therefore, the densely packed nanowire array template plays an instrumental role in enabling this type of MgO/ZnMgO nanowires [9]. Our analysis results pointed out that a nanoarray template is necessary for achieving solution processed alloying.

It is worth pointing out that the fabrication of ZnMgO semiconductor alloy nanorod array is to enable a new class of optoelectronic materials for light emitting diodes and visible range solar absorption using the low-cost low temperature solution process [8,9,35]. A low temperature (40 K) PL result shown in Figure 5a revealed comparative results of both ZnO (blue spectrum) and MgO/ZnMgO (red spectrum) nanowires. The low temperature (40 K) PL spectrum for ZnO nanowires revealed three peaks at 381 nm, 373 nm, and 391 nm as shown in the inset. These peaks are respectively due to the NBE peak responsible for the recombination of free excitons (381 nm), due to the ZnO seed layer on which the ZnO nanowires were grown (373 nm), and due to donor acceptor pair (DAP) (391 nm) as previously suggested by Park *et al.* [36]. Compared to ZnO nanowires, the NBE emission of MgO/ZnO nanowires is blue shifted to 367 nm from 381 nm, as shown in the inset of Figure 5b, which might be due to the modulation of the band gap caused by Mg substitution [37–39]. For both pure ZnO nanowires and as-grown MgO/ZnO nanowires, an NBE peak of 381 nm is revealed as a result of free exciton emission [40].

The observed blue shift of UV emission in the MgO/ZnO nanowires might be due to a successful Mg alloying into ZnO surface lattice, as an intermediate layer of ZnMgO between the dendritic amorphous MgO and ZnO core, leading to the widening of the energy band gap [41]. Furthermore, an enhancement in UV emission with a concomitant reduction in near-blue emission in MgO/ZnO nanowires, might suggest an improved crystallinity and a possible successful reduction of intrinsic defect (such as oxygen vacancy) concentration through the second-step 155 °C high-pressure hydrothermal “annealing” process. In addition, the surface amorphous layer of MgO could be beneficial for producing potential interface charge transfer events for enhancing the NBE UV emission [42].

It is clear that low temperature solution process can successfully introduce a certain amount of Mg alloying into ZnO lattice, while toward UV LEDs application, a larger alloying content may be necessary. Therefore, a post heat treatment study has been carried out. Two types of thermal annealing processes have been conducted including ambient and vacuum conditions [35]. Surprisingly, contrary to normal ambient annealing process in ZnMgO nanofilms, the ZnMgO nanowire ambient annealing at 900 °C for 5 min has led to complete quenching of NBE emission, while an enlarged and red-shifted (505 nm) defect-related band, as revealed in Figure 5b. During vacuum annealing, the defect-related band completely disappeared with an enhanced NBE emission (Figure 5c). The quenching of NBE band in ambient annealing could be attributed to the formation of (Zn,Mg)_1.7_SiO_4_ epitaxial layer. The strain introduced in the partially coherent core-shell interface lattice may provide a new design route for us to purposely enhance the visible absorption through defect generation and strained shell layer, which, in turn, could provide a very important strategy to design and enable good solar absorption materials. On the other hand, when ambient condition was changed to vacuum treatment, the formation of silicate will be much less likely and the defects in as-grown ZnMgO nanowires could be annihilated through suitable O_2_ partial pressure, which will help in decreasing the defect concentration, therefore visible emission intensity. The crystallinity and lattice perfection enhancement through this annealing process could have very important implications for light emission devices.

#### 2.2.2. Metal Oxide/Perovskite Composite Nanowires

The emergence of hierarchical nanostructures has stimulated various important practical applications in energy, environmental, electronic and optoelectronic sectors. Here in this section, we present our latest progress on the fabrication and utilization of metal oxide/perovskite (or spinel) composite nanowires for environmental catalysis and chemical sensing [10,11,13,14].

##### 2.2.2.1. ZnO/(La,Sr)CoO_3_ Nanowire Arrays for Photocatalysis and Photoresponsive Humidity Sensing

**Figure 6:**
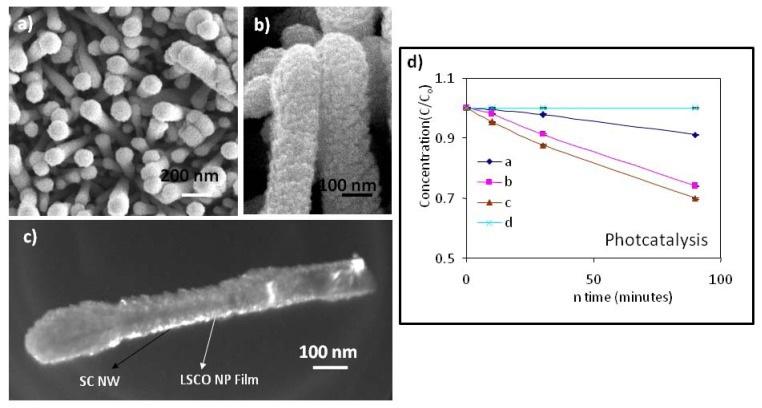
Top view (**a**) and side view (**b**) SEM images of ZnO/LSCO composite nanorod arrays based on ZnO nanorod arrays with a nanorod diameter of ~100 nm; (**c**) A dark field TEM image of an individual ZnO/LSCO composite nanorod indicating the bright regions corresponding to the dispersed LSCO nanoparticles with a grain size in a range of ~10–20 nm; (**d**) Photodegradation of methyl orange in the presence of (a) ZnO nanorod arrays, (b) LSCO thin film, (c) ZnO/LSCO composite nanorod arrays, and (d) a blank control.

By using a combination method of hydrothermal synthesis and pulsed laser deposition (or sol-gel deposition), we successfully fabricated ZnO/(La,Sr)CoO_3_ (LSCO) core-shell nanowire arrays, as shown in Figure 6 [6]. Top view and side view SEM images (Figure 6a,b), respectively) were recorded for ZnO/LSCO composite nanorod arrays based on ~100 nm thick ZnO nanorod arrays. The EDX spectrum analysis revealed that the LSCO/ZnO ratio in the nanocomposite based on the peak area ratio is about 1.55:1. The composite nanorod arrays were of rough grainy and mesoporous surface which was clearly seen in the SEM images and the dark field TEM images (Figure 6c). Similarly, ZnO/LSCO and TiO_2_/LSCO composite nanowire and nanodendrite arrays have been successfully grown using a combination method of hydrothermal synthesis and PLD deposition in our group.

It is worth noting that compared to the sol-gel process; the PLD process has given a uniform, more efficient and faster deposition of LSCO continuous film onto ZnO nanorod arrays. The sol-gel process instead, might provide a path for dispersed deposition onto the nanorod arrays, but with a much longer preparation time compared to a few-hour PLD deposition time. On the other hand, by decreasing the pulsed time and intensity, a thinner and mesoporous coating can be obtained. Furthermore, the deposited film uniformity was not just dependent on the PLD deposition parameters, but also dependent on the nanorod diameter, array density, and thus specific surface area of the nanorod arrays.

The photocatalytic properties of as-prepared PLD processed composite nanorod arrays were evaluated by measuring the absorption intensity of methyl orange at 464 nm and the absorption intensity of 4-chlorophenol (4-CP) at 262 nm. For both measurements, the excitation UV light wavelength was controlled at 254 nm. Both of these photodegradation reactions have been determined to be pseudo-first-order reactions, as evidenced by the linear photodegradation process as a function of irradiation time shown in Figure 6 (the methyl orange case). From Figure 6d, the reaction rate constants of methyl orange degradation were calculated for ZnO nanorods, LSCO film, and the corresponding ZnO/LSCO nanorods, which are 1.07 × 10^−3^ min^−1^, 2.90 × 10^−3^ min^−1^, and 3.18 × 10^−3^ min^−1^, respectively. Therefore, for the degradation of methyl orange (10 mg/L) using ZnO/LSCO nanorods sample (5 × 5 mm^2^), approximately 314 min are needed theoretically and actually after 470 min, 94% of the reaction is completed. By analogy, rate constants for the photocatalytic degradation reaction of 4-chlorophenol by ZnO/LSCO nanorods were deduced to be 1.22 × 10^−2^ min^−1^. For the degradation of 4-CP (6 mg/L) using ZnO/LSCO nanorods sample (5 × 5 mm^2^), approximately 82 min are required theoretically.

These photocatalysis data clearly demonstrate that composite nanorod arrays exhibited a higher photocatalytic activity as compared with those of both ZnO nanorod arrays and LSCO thin film samples. The observed enhancement of photocatalytic activity is most likely due to a relative increase in the active site concentration resulting from the ultra-high surface area in the mesoporous LSCO polycrystalline nanofilms and their interfaces with ZnO nanorods. To increase the photo-catalytic activity of the ZnO/LSCO composite nanoarchitecture, it is necessary to control the density and dimensionality of the packed ZnO nanorod arrays as a unique support structure, the LSCO thin film deposition parameters, the composition of LSCO and the interface structure design. These improvements would result in ZnO/LSCO composite nanoarchitectures with much better photocatalysis properties compared to ZnO nanorod arrays or monolithic monolayers of LSCO.

**Figure 7:**
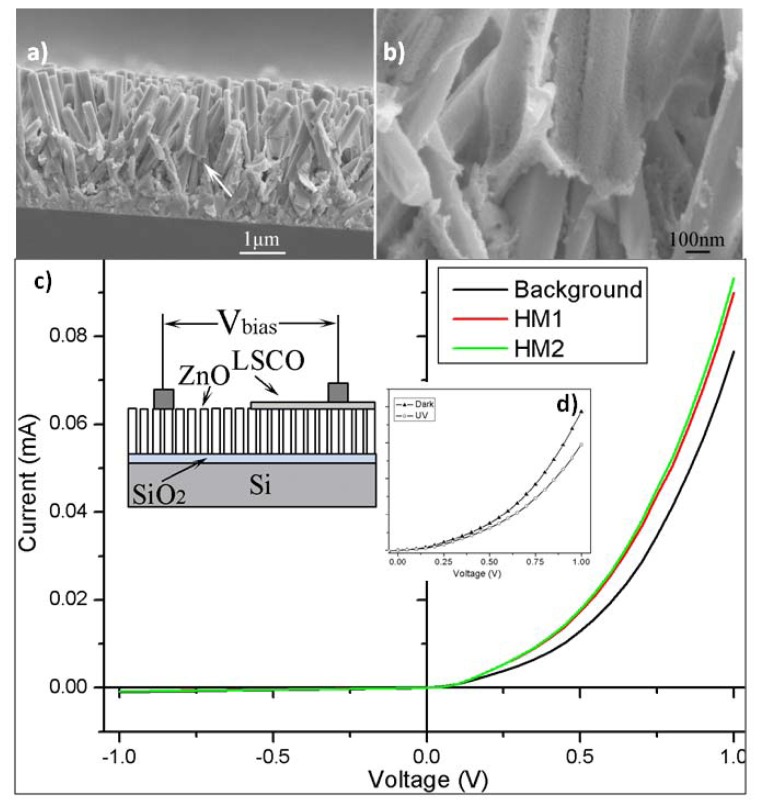
Low magnification (**a**) and high magnification (**b**) cross-sectional SEM images of ZnO/LSCO core-shell nanorods grown using a combination of hydrothermal and colloidal deposition processes; (**c**) Current-voltage (I-V) measurement of ZnO/LSCO heterostructure in the different humidity. The left inset shows the schematic illustration of the I-V measurement; (**d**) the decreased photocurrent response upon UV illumination compared to the dark.

Similarly, we have grown ZnO/LSCO core-shell nanorod arrays using a combination of hydrothermal and sol-gel process. Figure 7a,b show the cross-sectional SEM images of ZnO/LSCO composite nanorod arrays. From Figure 7a we can see that ZnO nanorods grew from the seed layer coated on Si substrate. The width of individual nanorod is very uniform from the tip to the bottom. From Figure 7b we can see that there are porous layers coated on ZnO nanorods. The LSCO materials were dispersed and coated on the surface of ZnO nanorods during the colloidal deposition.

To initiate the electrical characterization on the growth composite nanorod arrays, we started to look into the electronic transport property of ZnO/LSCO core-shell nanorods. The sample was baked at 80 °C overnight before a two-terminal current-voltage (I-V) property measurement. Figure 7c shows the I-V measurement of ZnO/LSCO heterostructure in the different humidity. The inset shows the schematic illustration of the I-V measurement across ZnO nanorod arrays and LSCO nanofilm. Here LSCO layer represents the LSCO nanofilm coated around ZnO nanorod arrays. A good rectifying characteristic have been found in the fabricated ZnO/LSCO heterostructure, which is similar to that of conventional semiconductor p-n junctions. The relative humidities of background, humidity 1 (HM 1) and humidity 2 (HM 2) were about 9%, 34% and 41%, respectively. The setting temperature of the hot plate was 80 °C. The slope of the I-V curve becomes larger under larger humidity circumstance as a result of decreasing forward resistance. X.Q. Fu *et al.* [43] reported the anomalous photoconductivity of CeO_2_ nanowires in air. With increasing humidity, H_2_O can be physically adsorbed on the nanostructures via hydrogen bonding. The nanostructure’s conductance then increases according to Grotthuss’s chain reaction, where proton transfer occurs among the hydronium [44]. So with the increasing humidity, the forward resistance of ZnO/LSCO heterostructure decreases.

##### 2.2.2.2. ZnO/(La,Sr)MnO_3_ Nanowire Arrays: Thickness Dependent Nanomagnetism

**Figure 8:**
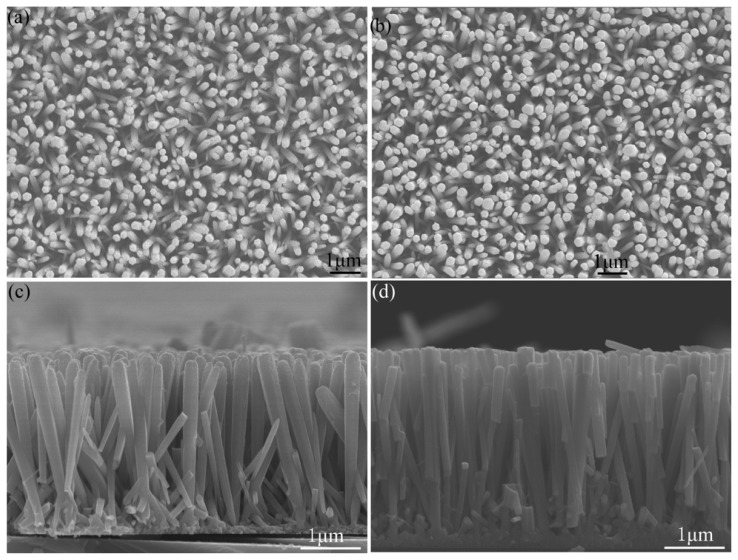
Top view SEM images of (**a**) as-grown ZnO nanorods and (**b**) LSMO coated on ZnO nanorods by sputtering. Cross sectional SEM image of LSMO coated around ZnO nanorods (**c**) by sputtering, and (**d**) by colloidal process.

ZnO/LSMO composite nanorod arrays have been synthesized through a two-step fabrication process on Si (100) flat substrates. Each composite nanorod is composed of a ZnO nanorod core and a LSMO nanoshell to enable ultra-high specific surface area. Figure 8a shows a typical top-view low-magnification SEM image of large-area ZnO nanorod array grown using hydrothermal method. A densely packed and well-aligned array of ZnO nanorods grew uniformly over the entire area. These nanorods grew along the [0001] direction as suggested by the hexagonal cross sections revealed from Figure 8a,b. The average diameter of the nanorods is ~100 nm. Figure 8b shows a top-view low-magnification SEM image of ZnO nanorod array after LSMO nanofilm deposition by sputtering. The morphology of LSMO on ZnO nanorods by colloidal deposition is very similar to that in Figure 8b. Comparing Figure 8a,b, large-area ZnO nanorod arrays and LSMO/ZnO nanorod arrays are clearly of similar morphology, which suggests that ZnO nanorods retain their well-aligned structure after LSMO nanofilm deposition. Figure 8c,d show the cross-sectional SEM images of LSMO/ZnO nanorod arrays deposited by sputtering and colloidal deposition, respectively.

It is clearly shown in Figure 8c,d that ZnO nanorods grew from the ZnO seed layer coated on Si substrate. The homogeneous coating of all the exposed parts of the nanorods has been achieved in both cases. The nanorods are aligned normal to the substrate despite a little randomness. From Figure 8c we can see that the width of individual nanorod is thinner and thinner from the nanorod tip to its bottom, which means the thickness of LSMO in the top part is larger than that in the bottom part. This is caused by the deposition process from the top of ZnO nanorod arrays with sputtering. The top part of individual nanorods looks rougher than the bottom part, which might be caused by different annealing effect of LSMO due to different thickness. While from Figure 8d we can see that the width of individual nanorod is fairly uniform from the nanorod tip to its bottom. Some nanorods are broken due to the cleavage process during the sample preparation.

**Figure 9:**
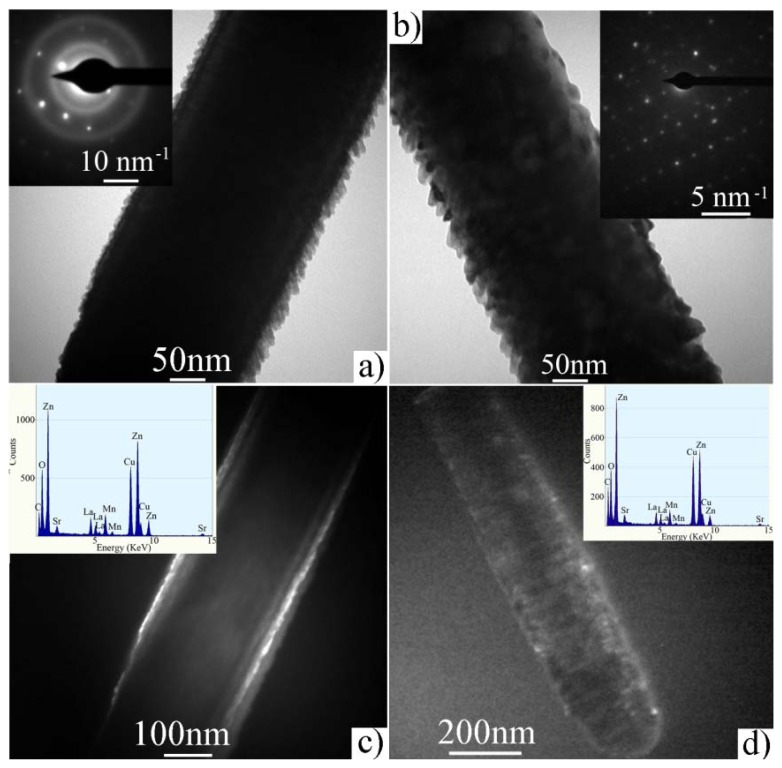
TEM image of as-deposited ZnO/LSMO composite nanorod: (**a**) and (**b**) bright field, (**c**) and (**d**) dark field. (**a**) and (**c**) correspond to as-deposited ZnO/LSMO, (**b**) and (**d**) correspond to annealed ZnO/LSMO. The insets in (**a**) and (**b**) show the corresponding electron diffraction pattern. The insets in (**c**) and (**d**) show the corresponding EDS spectrum.

Figure 9a,c displayed the bright field and dark field TEM images of a typical as-deposited ZnO/LSMO nanorod, respectively. Figure 9a clearly revealed the mesoporous rough surface of the nanorod. Inset in Figure 9a shows the SAED pattern of the as-deposited ZnO/LSMO nanorod. The diffusive ring pattern can be found besides the dot pattern that corresponds to ZnO single crystal. Therefore, the crystal structure of LSMO nanofilm coated on ZnO nanorod is polycrystalline but with poor crystallinity. In the dark field image shown in Figure 9c we can see clearly the core-shell structure of ZnO/LSMO nanorod. Figure 9b,d show the bright field and dark field TEM images of a thermally annealed ZnO/LSMO nanorod, respectively. The LSMO material nanofilm becomes smoother but dispersed after annealing, as indicated through a comparison of Figure 9a,b. From the SAED pattern of ZnO/LSMO shown in inset of Figure 9b, the dispersed LSMO nanofilm has very good crystallinity as revealed by scattered dotted pattern in addition to the single set of single crystal ZnO diffraction pattern, instead of a ring pattern. Figure 9d shows the dark field image of annealed ZnO/LSMO nanorod. Further characterization including high resolution TEM imaging and diffraction analysis are on-going to help further interpret the interface between the core and shell. The insets in Figure 9c,d are the corresponding EDS spectrums spectra to ZnO/LSMO nanorod before and after annealing, respectively. The peaks corresponding to O, Zn, La, Sr, and Mn were identified from both spectrums spectra. The Cu and C peaks come from the carbon-coated TEM Cu grid. The EDS spectra are similar for both as-deposited and annealed ZnO/LSMO composite nanorod.

**Figure 10:**
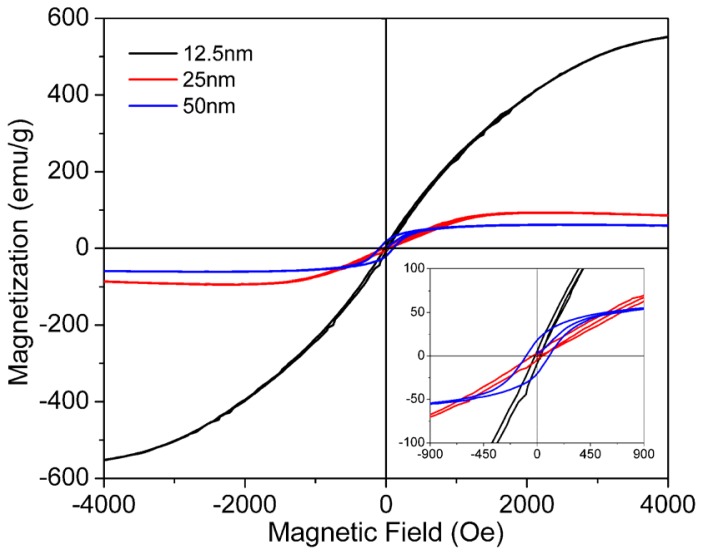
Typical M-H hysteresis curves of LSMO nanofilms with different thicknesses on 3D ZnO nanorod arrays measured at 80 K with magnetic field applied parallel to the film. Inset: the detailed M-H hysteresis curves at low magnetic field.

Magnetic property of LSMO has been suggested to be thickness dependent, including morphology, magnetoresistance and magnetization [45,46]. To investigate the thickness dependence of magnetization, LSMO nanofilms with various thicknesses have been studied on 3D ZnO nanorod arrays. Figure 10 shows the M-H hysteresis curve of LSMO nanofilm on ZnO nanorods with various thicknesses represented on flat Si. It is clear that the magnetization increases with the increase of magnetic field for LSMO with all thicknesses until saturation is reached. While the saturation magnetizations decrease with the increasing thickness of LSMO nanofilm, and the thinnest film has the largest saturation magnetization. If nanoparticles of LSMO are small enough, the single magnetic domains will interact with each other, leading to the superparamagnetism [47]. It is worth noting that the 12.5 nm is the thickness of LSMO on flat Si deposited at the same time, which suggests a real LSMO nanofilm on ZnO nanorod side surfaces is much thinner due to the increased surface area. Therefore, the ultrathin nanofilm of LSMO on 3D ZnO nanorod arrays shows the superparamagnetic property. The inset shows the zoom-in M-H hysteresis curves at low magnetic field. The hysteresis curves show that the curve area is nearly zero for 12.5 nm and 25 nm LSMO nanofilms, which indicates a large surface effect with too many Mn^3+^ ions near the surface to induce a superparamagnetic behavior. When the thickness becomes large, a well-behaved M-H curve was observed for the 50 nm LSMO nanofilm on ZnO nanorods substrate, indicating a ferromagnetic behavior of the LSMO nanofilm. In this case, the surface dispersion effect is relatively low, with anisotropic LSMO thin film exhibiting a ferromagnetic behavior like the LSMO nanofilm on 2D Si substrates.

##### 2.2.2.3. TiO_2_/(La,Sr)MnO_3_ Composite Nanowire Arrays for CO Oxidation

**Figure 11:**
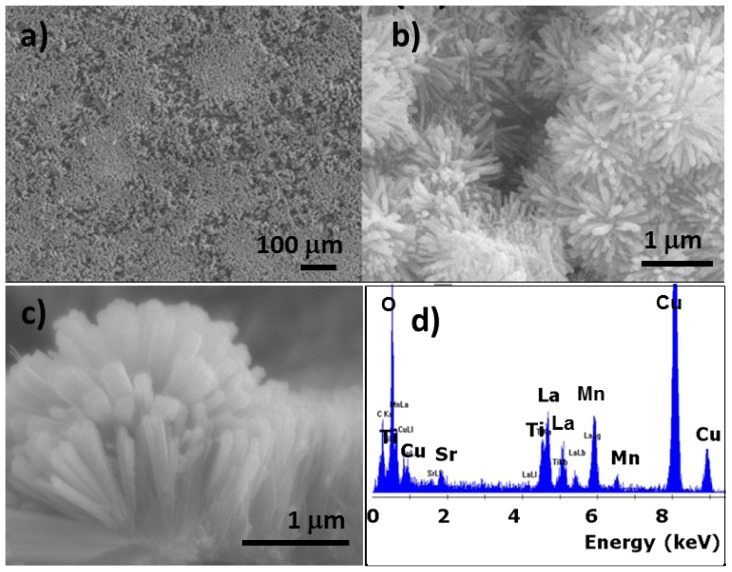
SEM images of TiO_2_/LSMO nanorod arrays: (**a**) large area image; (**b**) top-view of TiO_2_/LSMO nanorod arrays; (**c**) cross-view of synthesized nanorod array on silicon substrate; (**d**) EDS analysis under SEM.

**Figure 12:**
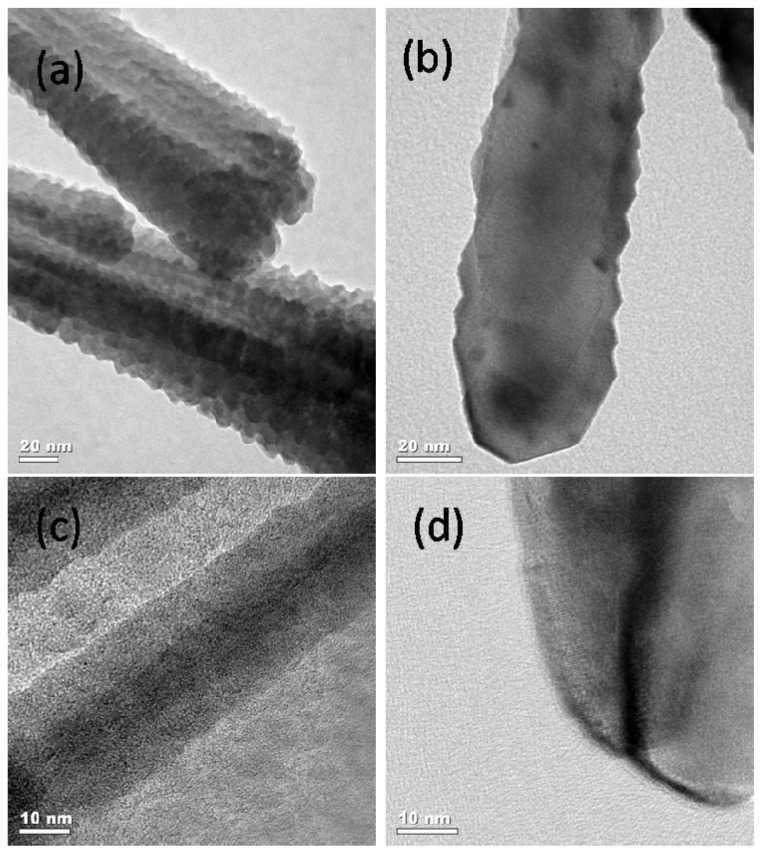
TEM images of the synthesized TiO_2_/LSMO nanorods with (**a**) and (**b**) before annealing; (**c**) and (**d**) after annealing at 800 °C for 3 h.

By using RF magnetron sputtering method, we fabricated TiO_2_/LSMO core-shell nanorod arrays on silicon substrate. First, we prepared TiO_2_ nanorod arrays on Si substrate by hydrothermal method. After that, we prepared TiO_2_/LSMO nanorod arrays from TiO_2_ nanorod arrays through RF magnetron sputtering LSMO nanofilm on the surface of TiO_2_ nanorod arrays. Figure 11a shows the large area TiO_2_/LSMO nanorod arrays and Figure 11b shows the top-view of TiO_2_/LSMO nanorod arrays on higher magnification. Figure 11c is the cross-view image of TiO_2_/LSMO nanorod arrays on silicon substrate. The TiO_2_/LSMO nanorods have diameters ranged from 30 nm to 70 nm and length of 1 μm. EDS results (Figure 11c) suggest the successful fabrication of TiO_2_/LSMO nanorods. By using TEM, we got more structure information about TiO_2_/LSMO nanorods. Figure 12a,c depict the TEM and HRTEM image of TiO_2_/LSMO nanorods before annealing respectively, while Figure 12b,d show the TEM and HRTEM image of TiO_2_/LSMO nanorods after annealing respectively. By annealing TiO_2_/LSMO nanorods at 800 °C for 3 h, the LSMO shell on TiO_2_ nanorod turns from amorphous into crystalline.

**Figure 13:**
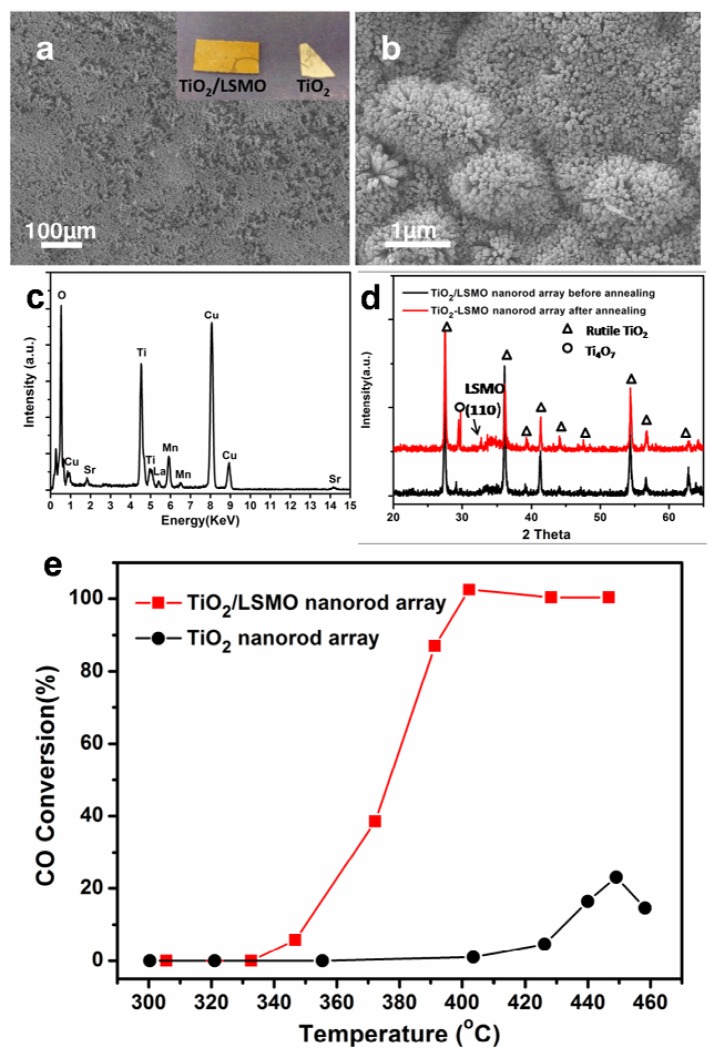
(**a**) a low magnification SEM top view of large area TiO_2_/LSMO nanorod arrays; inset: a photograph of Si substrates grown with TiO_2_/LSMO nanorod array and TiO_2_nanorod array, respectively; (**b**) a medium magnification top view of TiO_2_/LSMO nanorod array; (**c**) A typical energy-dispersive X-ray spectrum collected on TiO_2_/LSMO composite nanorod array; (**d**) Typical X-ray diffraction patterns (XRD) collected from as-synthesized composite nanorod array and 800 °C annealed TiO_2_/LSMO composite nanorod array; (**e**) CO oxidation conversion efficiency as a function of reaction temperature over the 800 °C annealed TiO_2_/LSMO composite nanorod array and TiO_2_ nanorod array.

Figure 13a,b shows the large area TiO_2_/LSMO nanorod arrays, which uniformly grow on Si planar substrate. The inset in Figure 13a depicts the photograph of Si substrate grown with TiO_2_/LSMO composite nanorod arrays and TiO_2_ nanorod arrays, respectively. The EDX analysis (Figure 13c) proved the successful fabrication of TiO_2_/LSMO composite nanorod array. The as-prepared TiO_2_/LSMO composite nanorod array does not exhibit perovskite LSMO peak due to the poorly crystallized as-deposited LSMO shell. While after 800 °C ambient annealing, the XRD pattern of TiO_2_/LSMO composite nanorod array does display an obvious peak at 32.9° as indicated by an arrow in Figure 13d, which was identified as (110) atomic planes of cubic perovskite La_0.8_Sr_0.2_MnO_3_ (JCPDS# 04–006–9331, SG: Pm−3m (221), *a* = 3.86 Å), indicating that the LSMO film might be dominated by (110) oriented facets. In the meantime, we also investigated the Nitrogen adsorption BET specific surface area of 800 °C annealed TiO_2_/LSMO composite nanorod array and TiO_2_ nanorod array on Si substrate respectively (Table 1). The measured BET specific surface area of TiO_2_/LSMO nanorod array on Si substrate decreased for nearly 25%, which may be because the space between nanorods were decreased after sputtering LSMO nanoshell. To evaluate the catalytic performance TiO_2_/LSMO composite nanorod array, we select CO oxidation reaction as probe reaction. Catalytic CO oxidation was performed over the 800 °C annealed TiO_2_/LSMO composite nanorod array and TiO_2_ nanorod array on Si substrate separately. Figure 13e shows temperature programmed CO oxidation conversion curves of the TiO_2_/LSMO composite nanorod array and individual TiO_2_ nanorod array, respectively. The onset oxidation temperature of TiO_2_/LSMO composite nanorod array is 330 °C, about 90 °C lower than that of TiO_2_ nanorod array in 420 °C. The half conversion temperature and oxidation plateau temperature of 375 °C and 400 °C for the TiO_2_/LSMO composite nanorod array are also 50–60 °C lower than those of the TiO_2_ nanorod array (~435 °C and ~450 °C). Moreover, compared to the low maximum CO conversion efficiency of TiO_2_ nanorod array (~20%), TiO_2_/LSMO composite nanorod array achieved 100% CO conversion efficiency at 400 °C. The complete conversion temperature of TiO_2_/LSMO composite nanorod array is also lower than reported perovskite (La_0.5_Sr_0.5_MnO_3_) powder. It can be found from the above CO oxidation analysis that the introduction of LSMO promotes the CO oxidation greatly.

#### 2.2.3. Metal Oxide (Ag_2_O)/Spinel (Zn_2_SnO_4_) Composite Nanowires

**Figure 14:**
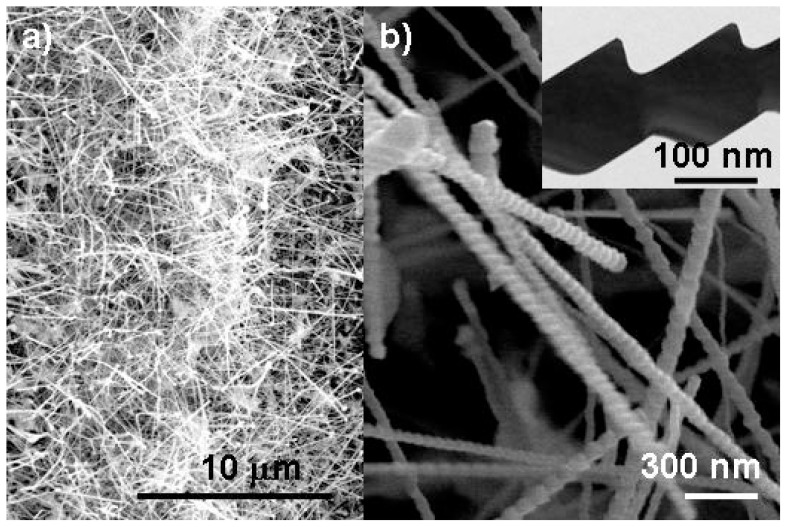
The SEM images of single crystalline Zn_2_SnO_4_ nanowires.

Spinel structured single crystalline Zn_2_SnO_4_ nanowires have been synthesized using an oxide-assisted chemical vapor deposition for ethanol detection at 150 °C [12]. Figure 14 is a set of scanning electron microscopy (SEM) images showing the grown ZTO nanowires on SiO_2_/Si substrate. A high yield of nanowires grew uniformly in a diameter of ~50–200 nm and a length up to tens of micrometers on the substrate (Figure 14a). These zigzag structured nanowires were composed of chaining trapezoid shape unit blocks with depth of each around 50 to 200 nm (Figure 14b).

**Figure 15:**
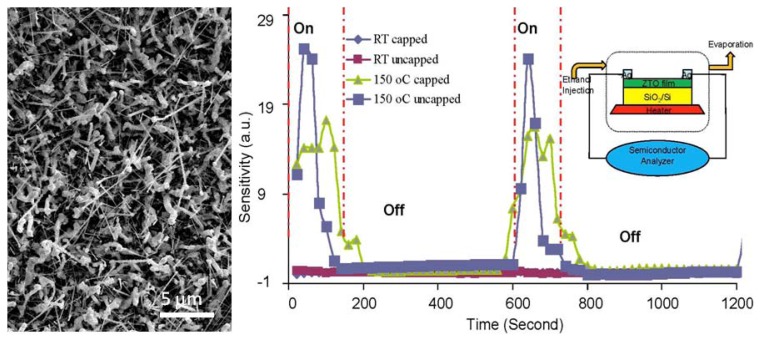
SEM image of top view Zn_2_SnO_4_ (ZTO) periodic nanowires (**left**), and (**right**) the time dependence sensitivity spectra of ZTO nanowire devices in both open (uncapped) and close (capped) atmosphere at room temperature and 150 °C.

To explore the gas sensing performance of Ag_2_O/Zn_2_SnO_4_ (ZTO) hybrid nanowires, the ethanol sensing property was studied based on open and close atmospheres with a ~150 ppm ethanol pulse “on” and “off” every 10 min. Figure 15 shows a typical time-dependence resistance spectrum in the fabricated ZTO nanowire ethanol sensors. The input ethanol pulse did not change the resistance of ZTO nanowire film appreciably in either open or close environments at room temperature. At temperature of 150 °C, the ZTO nanowire film resistance increased an order of magnitude upon ethanol input, giving rise to a high sensitivity of ~18–28 in open and closed environments which is comparable to or better than the other prevalent gas sensor nanomaterials like SnO_2_ and ZnO [48–50]. The input reducing atmosphere (150 ppm ethanol pulse) actually activated the sensitivity of nanowire film over the dominant ambient oxygen in a very short rise time (~40 s and ~80 s, respectively in open and close environments, as shown in Figure 15. The 150 ppm ethanol pulse helped to free a large number of surface donors due to the bounded ambient oxygen molecules at 150 °C, which therefore activated the metal oxide surface sensing activity to the ambient oxygen. Furthermore, the conversion from incompletely consumed and discontinuous Ag_2_O to Ag in the presence of reducing ethanol could be another explanation [51]. This reduction could lead to the presence of Ag nanoparticles, which is a typical oxidation catalyst by speeding the dissolution of oxygen molecules onto the bounded surfaces.

## 3. Thermally Engineered Composite (Heterogeneous) Nanoarchitectures

### 3.1. Metal Hydroxystannates and the Derivative Stannate Composite Nanostructures

Zinc Hydroxystannate (ZHS) is known as one of the most promising flame retardants and smoke suppressants used in a wide array of engineering materials including plastics, rubber, paint, and other polymeric systems. The superior degradation resistance of ZHS to fire and flame originates from its highly endothermal phase transition to zinc stannate (ZS). The perovskite structured ZS, a ~1 eV narrow band gap semiconductor in theory [52], possesses non-linear optical, ferroelectric and piezoelectric properties, making it a potentially important multi-functional material for clean and high efficiency renewable energy applications. However, the ZS crystalline phase forms normally at very high pressure above 7 GPa [53,54]. Its fundamental properties have been largely unexplored to date. Heat-treating ZS at elevated temperatures between ~600 °C and 800 °C will decompose it into tin oxide (TO) and spinel zinc stannate (SZS), which have shown great promise in photovoltaic and transparent conductive electrode applications.

**Figure 16:**
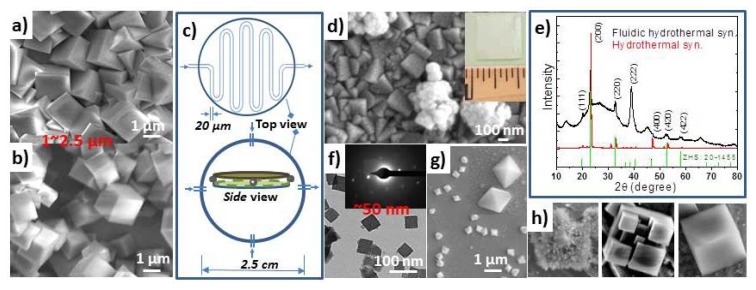
Single crystalline ZHS cubes grown on pure tin substrate (**a**) and ITO/quartz substrate (**b**) via a seedless static hydrothermal synthesis method; (**c**) A typical 1” fluidic cells introduced to the conventional hydrothermal method for improving the yield and uniformity of ZHS cube films. (**d**) Continuous ZHS cube films on ITO/quartz by fluidic synthesis, inset: 1 cm by 1 cm ZHS coated slide. (**e**) Multiple-facet ZHS nanoparticle films grown by fluidic synthesis *versus* {200}-facet ZHS cube film from conventional hydrothermal method, revealed by their corresponding XRD spectra. (**f**–**h**) Nanosized and different morphological ZHS cubes obtained by fluidic hydrothermal deposition.

In the past, ZHS, ZS or SZS particles have been obtained via various wet chemical methods such as co-precipitation, ion-exchange reaction, and sol-gel, and physical vapor method including, thermal evaporation, calcination, and mechanical grinding. Recently, our effort is devoted to the *in situ* scale-up synthesis of stannate nanomaterials on metal, fabrics, Si, and transparent conducting substrates [15–17]. The synthesis of size-tunable, density-controlled and conformal single crystalline ZHS cubes on Sn metal substrates was achieved via a seedless, low temperature (60 °C) hydrothermal synthesis approach (Figure 16a,b). This method exhibits low energy consumption (60 °C oven for as little as 2 h), but still yields highly robust ZHS coatings. However, the conventional hydrothermal approach did not work as well on transparent conducting substrates. To improve the control of density of the ZHS nanostructures on various substrates, we introduced the fluidic cells to the hydrothermal synthesis process (Figure 16c). 1 cm × 1 cm dense and continuous ZHS cube films have been achieved on both metal and transparent conducting substrates, such as ITO/quartz and SnO_2_/quartz (Figure 16d).

**Figure 17:**
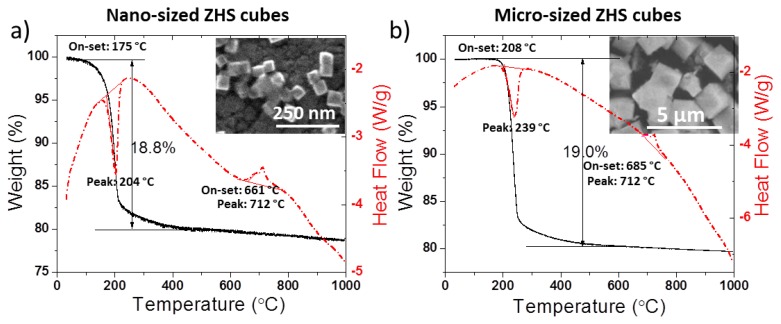
SEM image and TGA/DSC curves for nano-sized ZHS cubes (**a**) and micro-sized ZHS cubes (**b**).

**Figure 18:**
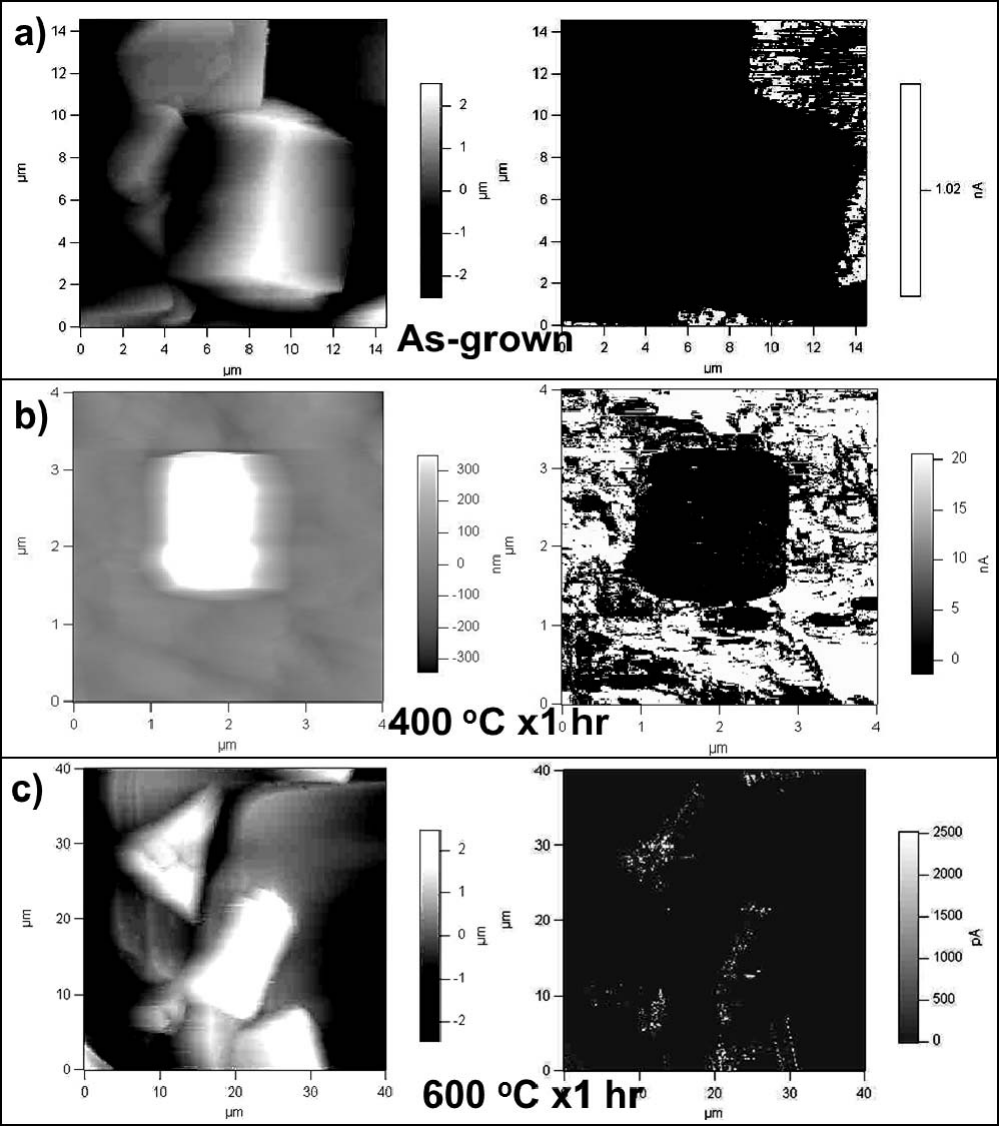
Initial atomic force microscopy micrographs obtained on ZHS cube surfaces after thermal annealing at different temperatures: (**left**) topography image; (**right**) conductivity map corresponding to the topography image.

**Figure 19:**
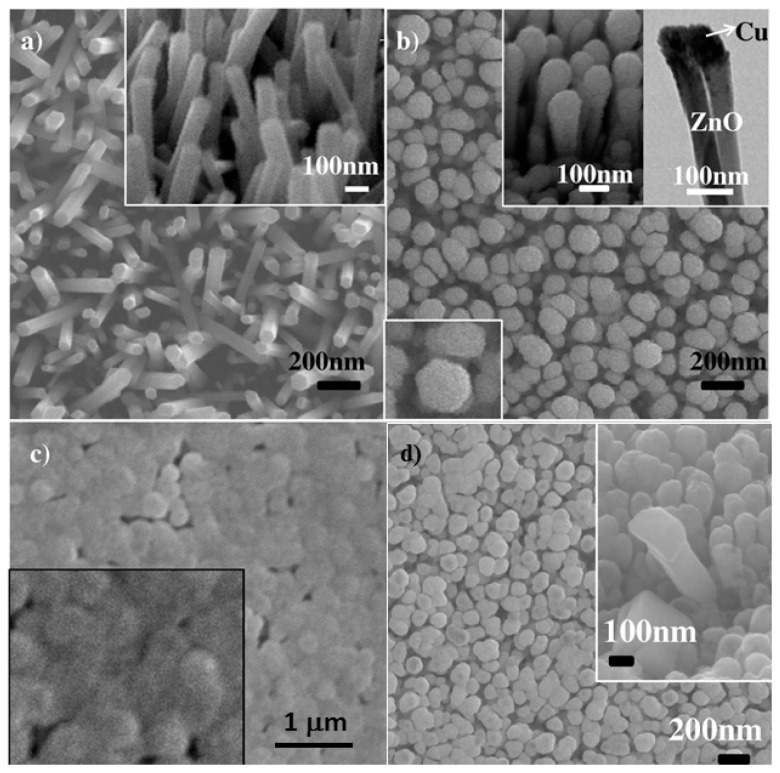
Cu film oxidation on ZnO nanowire arrays: Top view SEM images (**a**) ZnO nanorods grown by hydrothermal method, inset: a 30° tilt view of nanorods; (**b**) ZnO-Cu core-shell nanorods after deposition of Cu and upper right inset (left) showing their nail-shaped structure, upper right inset (right): TEM image of ZnO-Cu core-shell nanorods; bottom left inset: zoom in view of a hexagon shaped nanorod tip; (**c**) ZnO-Cu core-shell nanorods annealed at 400 °C in ambient air for 30 min, inset: zoom in view of “flat” and continuous CuO film bridging adjacent ZnO nanrods; (**d**) Top view SEM image of ZnO-CuO core-shell nanowire arrays: 80 sccm oxygen flow rate and 100 mabr pressure.

**Figure 20:**
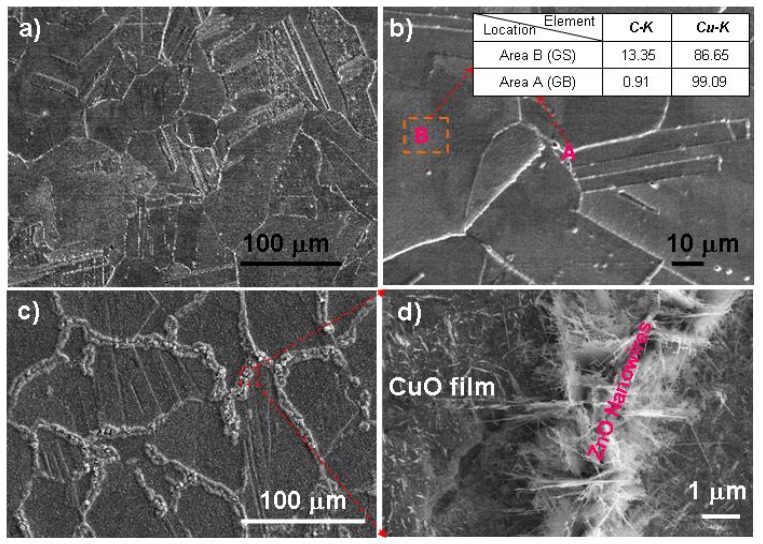
(**a**,**b**) are respectively, a low-magnification and a high-magnification SEM image of metallographically etched Cu_70_Zn_30_ polycrystalline substrate revealing clearly the GB networks. Inset in (**b**) is an EDXS composition table revealing the Cu-rich GS after etching process; (**c**) A typical low-magnification top view SEM image of ZnO nanowires/CuO film heterojunction network after 4-h ambient oxidation at 500 °C; (**d**) A zoom-in top view SEM image showing the distinct growth morphology in the GS region (CuO nanofilms) and GB region (ZnO nanowires).

Each ZnSn(OH)_6_ cube is enclosed by six equivalent {001} crystal planes [14]. The cube size can be adjusted from 50 nm (Figure 16f) to 20 μm. The cube aerial density can be varied from sparse to continuous film by controlling reaction time and concentration of diaminopropane (DAP) reagent. The hexamethylenetetramine (HMT) and DAP assisted etching of the oxidized tin surface was found to play an important role in the nucleation and growth of the zinc hydroxystannate cubes on tin foil substrate. The research on further understanding the formation mechanism of ZHS nanostructures is undergoing. ZHS cube film synthesized via fluidic hydrothermal approach show much larger ratio of high index facet than the one obtained from conventional method. Figure 16e is the XRD spectra for ZHS films. The (222) signal shows much higher relative intensity in fluidic sample than that of the hydrothermal method. Other interesting stannate structure also can be synthesized via fluidic hydrothermal method, such as the nanomaterials illustrated in Figure 16g,h.

While there has been some success on synthesis of ZHS powders, the thermal decomposition process has not been thoroughly researched to date. A rough decomposition temperature region has been estimated at ~200–380 °C for bulk sample. However, studies relating decomposition process to structural and morphological transformations within micron and nano-sized ZHS crystallite are not available. This information, if available, would help control the film structure evolution, allowing for the fabrication process optimization. To obtain this valuable information, slow equilibrium and fast non-equilibrium thermal annealed ZHS cube films have been investigated using electron microscopy (EM), thermal gravimetric analysis (TGA), differential scanning calorimetry (DSC), X-ray diffraction (XRD), and conductive Atomic Force Microscopy (CAFM).

We have studied the structure-property evolution study of the as-fabricated ZHS micro- and nano-cubes after different thermal annealing processes. TGA/DSC analysis for micro- and nano-sized ZHS (Figure 17) suggested that two phase transitions occur during ZHS thermal annealing at around 208 °C and 685 °C for the 1~2.5 micron ZHS cubes. While, for the 50 nm ZHS cubes, the decomposition starts at 20 °C earlier. On the other hand, it was interesting to find out that the ZHS cube shape remained intact following 2 h thermal annealing at 600 °C (inset of Figure 17a). The only noticeable change was surface roughening. The appearance of endothermic peak near ~204 °C (Figure 17a), attributed to the zinc hydroxystannate decomposition/dehydration, was correlated with morphology changes induced via resistive thermal annealing and localized electron beam heating.

Topography AFM and Conductive AFM have been used to unravel the morphology-structure-property evolution of ZHS cubes after thermal annealing in an ambient atmosphere. Figure 18 illustrates the AFM and CAFM micrographs obtained on ZHS cube film surfaces before and after thermal annealing. The surfaces of as grown ZHS cubes appear very smooth with no measurable current from the CAFM tip through the cubes. These results point to highly insulating ZHS cubes characterized by the energy band gap of ~7 eV. Meanwhile, slight current leakage from the tip to the cubes was observed with the 400 °C annealed sample (*i.e.*, top left corner of the cube in the rightmost Figure 18b image). This suggests formation of semiconductive nanocrystals (potentially covered by ZS, as evidenced by XRD analysis). Increasing the annealing temperature to 600 °C resulted in more leakage current traces as evidenced by Figure 18c CAFM micrograph. This indicates formation of more semiconductive phases due to structure conversion from ZHS to partial ZHS/ZS.

### 3.2. Radial Heterojunction and Heterojunction Network of Metal Oxide Semiconductors

The engineering and enabling of heterojunction between two dissimilar semiconductors have guided the development and evolution of semiconductor industry. At nanoscale, it is a key to have a respectively scalable and economic way to produce the counterpart heterojunction in nanoscale. However, it undoubtedly presents a new challenge given the space confined fabrication and operation in heterojunction formation coming into play. Here we have taken the interface junction between CuO and ZnO at nanoscale as an example to summarize our latest progress along the heterojunction formation and patterning effort using a simple thermal oxidation strategy. Utilizing two kinds of template oxidation models, *i.e.*, Cu nanofilm on ZnO nanowire arrays [18] and dezincified heterogeneous compositional surface (Cu-rich surfaces and Zn-rich grain boundaries) of Zn-Cu alloy [19], we successfully enabled both ZnO-CuO core-shell nanowire arrays and ZnO-CuO heterojunction networks using a metallurgically patterned surface of Zn-Cu alloy.

To form the axial core-shell ZnO-CuO heterojunction nanowire arrays, we grew ZnO nanowire arrays on ZnO seeded Si or quartz substrates, followed by sputtering of Cu to enable ZnO-Cu core-shell heterojunction templates, which are then thermally oxidized under controlled oxidative atmosphere. Illustrated in Figure 19 is the thermally oxidized Cu surface on the three-dimensional ZnO nanowire arrays. Figure 19a shows a typical top view SEM image of the as-grown ZnO nanorods on 50 nm ZnO-seeded Si (100) substrate after hydrothermal process. Densely packed and uniformly aligned ZnO nanorod arrays have been fabricated with a diameter of ~80–100 nm, and a length of ~500–1000 nm on the substrate, as revealed by 30° tilt view SEM image shown in the inset. After a 20 nm Cu sputtering deposition on 3D ZnO nanorod arrays, the nanorod diameter became ~100–120 nm, while the resulted ZnO-Cu core-shell nanorods retained their shape, as revealed in Figure 19b. The gap between adjacent ZnO-Cu nanorods is ~10–50 nm and 45° tilt view SEM image and the TEM image shown in the upper right inset of Figure 19b suggests that Cu has been deposited onto ZnO nanorods as a “nanofilm”, leading to a nail-shaped nanorod. EDX spectrum analysis revealed ~16.27 at.% of Cu present in the top portion of ZnO-Cu nanorods, while drastically decreased Cu content of ~6.91 at.% in the bottom portion of ZnO-Cu nanorods. This further suggests that Cu intends to accumulate on top of ZnO nanorods resulting in the nail-shaped core-shell structure.

To convert the ZnO-Cu nail-shaped core-shell nanorod arrays into ZnO-CuO core-shell nanorod arrays, ambient oxidation at 400 °C was carried out for 1 h, which, however, turned into nearly solid “flat” film on ZnO nanorod arrays as shown in Figure 19c. The formed film was confirmed as CuO by EDX and X-ray diffraction analyses which are consistent with the recent report. With the small gap of 10–50 nm between adjacent ZnO-Cu nanorods, it is likely that rapid oxidation process happened in ambient condition (1 bar), leading to a quick outward top surface oxidation with enough oxide film fill in the nanorod gaps. To avoid the fast oxidation process and improve the conformality of CuO film formation on the 3D nanorod arrays, a systematic study of the low pressure (<1 bar) and oxygen flow rate dependence has been carried out on the thermal oxidation behavior of Cu nanofilm on the 3D ZnO nanorod arrays. The Table 2 summarizes the various investigated samples after different thermal oxidation treatments. Figure 19d shows a top-view SEM image of ZnO-CuO core-shell nanorods formed with an oxygen flow of 80 sccm under 100 mbar. The comparison of these top view images suggested that with increasing oxygen flow rate, thin-film like Cu*_x_*O morphology is more likely to form surrounding ZnO nanorods. Early studies have suggested that variation of oxygen partial pressure during thermal oxidation could affect the morphology of copper oxide film [55,56]. At high pressure, fast Cu*_x_*O substances fill-in tended to occur between adjacent ZnO-Cu nanorods, leading to drastically increasing barrier of the oxygen diffusion from the top portion to the bottom portion of nanorod arrays. This will lead to the dominant upward (↑) oxidation of Cu film, forming a relatively flat CuO nanofilm on top of ZnO nanorod arrays. While lower pressure could help enable a more conformal CuO nanofilm formation on the three-dimensional ZnO nanowire array surfaces.

While for ZnO-CuO heterojunction network, we use the surface etched Cu-Zn polycrystalline alloy, which is treated in a controlled oxidative atmosphere during thermal oxidation. On the other hand, by directly heating a surface-dezincified single phase polycrystalline brass (Cu_70_Zn_30_ alloy) in ambient conditions, we successfully fabricated semiconductor nanostructure networks composed of CuO nanofilm and ZnO nanowires [18]. The dezincification induced by metallographic etching on Cu_70_Zn_30_ polycrystalline alloy surface has been used to create heterogeneous networks composed of individual Cu-rich micro-grain surfaces (GS) and surrounding grain boundaries (GB), as shown in Figure 20a,b. The comparative thermal oxidation results evidence distinct semiconductor nanowire/nanofilm network junctions on dezincified Cu_70_Zn_30_ substrates compared to the random ZnO nanowires growth on non-etched brass substrates, as illustrated in Figure 20c,d. The grain boundary region is mostly dominated by ZnO nanowires, while each grain surface was overgrown with CuO nanofilm, forming nanowire/nanofilm network junctions.

The different growth habits of ZnO nanowires and CuO nanofilms are due to the composition distribution difference between the dezincified Cu_70_Zn_30_ grain surface and grain boundary, resulting in heterogeneous diffusion and oxidation kinetics of Cu and Zn element on the grain surfaces and the grain boundaries. We believe, the surface dezincification and selective oxidation process in single-phase or more complex multi-phase Cu-Zn alloy substrates could provide a new approach for heterogeneous particulate nanostructure design, fabrication and control. The heterogeneous semiconductor nanowire/film network junctions may be used as nanoscale building block for electronic and optoelectronic devices.

## 4. Conclusions

This review mainly highlights our latest work on metal oxide-based composite nanostructures, specifically in semiconductor oxide alloy nanowires, metal oxide/perovskite composite nanowires, stannate based nanostructured composites, and ZnO-CuO heterojunction nanowire arrays and surface networks, which could impact the application sectors in ultraviolet/blue lighting, visible solar absorption, vehicle and industry emission control, chemical sensing and control for transportation, energy production, and environmental protection. By utilization of the template strategy in conjunction with various synthetic approaches including the wet chemical process, vapor phase deposition, and thermal engineering, or a combination method, various oxide-based nanostructures in hierarchical fashion, such as nano-arrays and networks, can be rationally achieved.

Specifically, first, by using a low temperature 2-step sequential hydrothermal process, Mg alloying in ZnO nanorod arrays has been successfully achieved, which established a unique low-cost alloying/doping process for semiconductor nanowires. On the other hand, through different atmosphere annealing control, the NBE emission could be either quenched with red-shifted and enlarged defect-related visible band, or enhanced with a complete quenching of defect-related band, which respectively would have impacts on visible solar absorption or UV LED. Second, ZnO/LSCO, ZnO/LSMO, TiO_2_/LSMO, and Ag_2_O/Zn_2_SnO_4_ composite nanorod arrays have been synthesized through a combination of hydrothermal, colloidal deposition, or physical vapor deposition. These composite nanorod arrays have been demonstrated to have excellent photocatalytic degradation over organic dyes, photoresponsive humidity detection ability, good CO oxidation performance, thickness dependent anisotropic magnetic properties, as well as the reversible catalytic ambient oxygen/ethanol detection ability. Third, on stannate based nanostructured composites, a universal precursor was used from hydroxystannate compound based nanostructure, which could be actualized into various heterogeneous stannate based nanostructures through rational control of the thermal decomposition process. The control of the thermal decomposition could consist of a few important parameters such as heating ramping rates, duration, atmosphere, *etc*. Finally, through thermal oxidation temperature control, pressure and oxygen flow, ZnO-Cu core-shell nanowire arrays could be converted into conformal core-shell heterojunction arrays in ZnO-CuO. While the surface metallurgically patterned microstructures could be directly thermally oxidized into ZnO-CuO nanowire-nanofilm heterojunction networks. These demonstrations may lead to a new array of advanced nanotechnologies in solid state lighting, solar absorption, photocatalysis and battery, auto-emission control, and chemical sensing.

## Figures and Tables

**Schematic 1:**
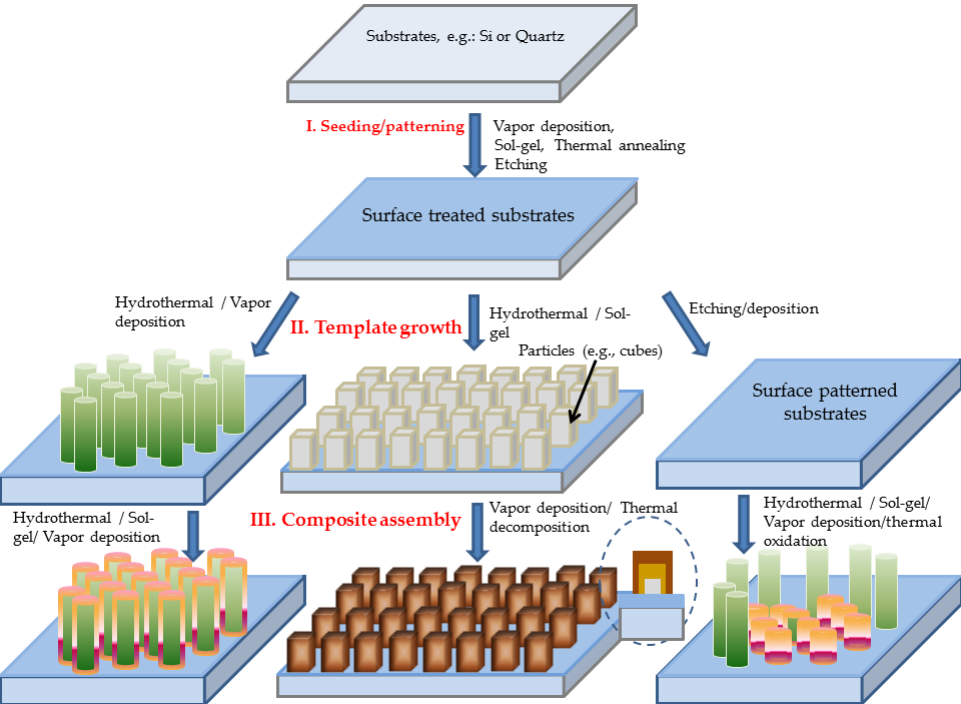
The composite nanostructure fabrication strategy flow chart.

**Table 1:** Multi-point nitrogen adsorption BET specific surface area of TiO_2_ nanorod array and 800 °C annealed TiO_2_/LSMO composite nanorod array grown on Si substrates.

Sample	Measured BET specific surface area (m^2^/g) including substrate	Estimated BET specific surface area (m^2^/g) without substrate
TiO_2_ nanorods on Si substrate	2.07	19.4
TiO2/LSMO composite nanorods on Si substrate	1.58	13.9

**Table 2:** A list of five typical ZnO-CuO core-shell nanorod array samples (S0, S1, S2, S3, and S4) formed after various thermal oxidation processes with different pressure and oxygen flow rate control.

Parameters	Temperature (°C)	Pressure (mbar)	Oxygen flow (sccm)	Core-shell characteristic

Samples				
S0	400	100	0	Poor/non-conformal
S1	400	100	20	Poor/non-conformal
S2	400	100	50	Good/conformal
S3	400	100	80	Ok/less-conformal
S4	400	500	50	Poor/non-conformal

## References

[1] Gao P.X., Umar A. (2010). Metal-catalyzed nanoarchitectures of zinc oxide and their applications. Metal Oxide Nanostructures and Their Applications.

[2] Lee J.H. (2009). Gas sensors using hierarchical and hollow oxide nanostructures: Overview. Sens. Actuators B Chem.

[3] Cai W.J., Gao H.Y., Kim D.S., Gao P.X., Umar A. (2012). Hierarchical Self-assembly of Semiconductor Nanowires for Electronics, Energy and Environment Applications. Encyclopedia of Semiconductor Nanotechnology.

[4] Moore D., Ding Y., Wang Z.L. (2006). Hierarchical structured nanohelices of ZnS. Angew. Chem. Inter. Ed.

[5] Miszta K., de Graaf J., Bertoni G., Dorfs D., Brescia R., Marras S., Ceseracciu L., Cingolani R., van Roij R., Dijkstra M. (2011). Hierarchical self-assembly of suspended branched colloidal nanocrystals into superlattice structures. Nat. Mater.

[6] Baibich M.N., Broto J.M., Fert A., Nguyen van Dau F., Petroff F., Eitenne P., Creuzet G., Friederich A., Chazelas J. (1988). Giant magnetoresistance of (001)Fe/(001)Cr magnetic superlattices. Phys. Rev. Lett.

[7] Binasch G., Grünberg P., Saurenbach F., Zinn W. (1989). Enhanced magnetoresistance in layered magnetic structures with antiferromagnetic interlayer exchange. Phys. Rev. B.

[8] Shimpi P., Ding Y., Surez E., Ayers J., Gao P.X. (2010). Annealing induced nanostructure and photoluminescence property evolution in solution-processed Mg-alloyed ZnO nanowires. Appl. Phys. Lett..

[9] Shimpi P., Gao P.X., Goberman D., Ding Y (2009). Low temperature synthesis and characterization of MgO/ZnO composite nanowire arrays. Nanotechnology.

[10] Jian D.L., Gao P.X., Cai W.J., Allimi B.S., Alpay S.P., Ding Y., Wang Z.L., Brooks C. (2009). Synthesis, characterization, and photocatalytic properties of ZnO/(La,Sr)CoO_3_ composite nanorod arrays. J. Mater. Chem.

[11] Gao H.Y., Cai W.J., Shimpi P., Lin H.-J., Gao P.X. (2010). (La,Sr)CoO_3_/ZnO nanofilm-nanorod diode arrays for photo-responsive moisture and humidity detection. J. Phys. D Appl. Phys.

[12] Cai W.J., Shimpi P., Jian D.L., Gao P.X. (2010). Oxide-catalyzed growth of Ag_2_O/Zn_2_SnO_4_ hybrid nanowires and their reversible catalytic ambient ethanol/oxygen detection. J. Mater. Chem.

[13] Gao H.Y., Staruch M., Jain M., Gao P.X., Shimpi P., Guo Y.B., Cai W.J., Lin H.-J. (2011). Structure and magnetic properties of three-dimensional (La,Sr)MnO_3_ nanofilms on ZnO nanorod arrays. Appl. Phys. Lett.

[14] Guo Y.B., Zhang Z.H., Gao H.Y., Ren Z., Gao P.X. (2012). Synthesis and characterization of TiO_2_/(La,Sr)MnO_3_ composite nanostructures as CO oxidation catalysts. Catal. Today.

[15] Wrobel G., Piech M., Dardona S., Ding Y., Gao P.X. (2009). Seedless synthesis and thermal decomposition of single crystalline zinc hydroxystannate cubes. Cryst. Growth Des.

[16] Wrobel G., Piech M., Dardona S., Gao P.X. (2012). Synthesis and fire retardant property of zinc hydroxystannate coated microfibers. Sci. Adv. Mater.

[17] Liu C.H., Chen H.Y., Wrobel G., Guo Y.B., Dardona S., Piech M., Bai J.M., Shao M., Gao P.X. (2012). Thermally tuning the structure, optical and photocatalytic properties in versatile nanostructured stannate composites: A case on zinc hydroxystannate.

[18] Liao K.-T., Shimpi P., Gao P.X. (2011). Thermal oxidation of Cu nanofilm on three dimensional ZnO nanorod arrays. J. Mater. Chem.

[19] Sarac M.F., Shimpi P., Mackey J.A., Kim D.S., Gao P.X. (2010). Surface dezincification and selective oxidation induced heterogeneous semiconductor nanowire/nanofilm network junctions. Cryst. Growth Des.

[20] Gao P.X., Song J.H., Liu J., Wang Z.L. (2007). Nanowire piezoelectric nanogenerators on plastic substrates as flexible power source for nanodevices. Adv. Mater.

[21] Guo Y.B., Ren Z., Zhang Z.H., Gao P.X. (2012). Ultra-efficient, robust and well-defined nano-array based catalysts.

[22] Zhang Z.H., Gao H.Y., Cai W.J., Liu C.H., Guo Y.B., Gao P.X. (2012). *In situ* TPR removal: A generic method for fabricating tubular structure array devices with mechanical and structural soundness, and function robustness on various substrates.

[23] Lin H.J., Gao H.Y., Gao P.X. (2012). Unpublished results.

[24] Wang X.D., Gao P.X., Li J., Summers C.J., Wang Z.L. (2002). Rectangular porous ZnO-ZnS nanocables and ZnS nanotubes. Adv. Mater.

[25] Shimpi P., Gao P.X. (2010). Carbon assisted and strain driven lateral alignment of silica nanowires. Cryst. Eng. Commun.

[26] Zhang H.Z., Kong Y.C., Wang Y.Z., Du X., Bai Z.G., Wang J.J., Yu D.P., Ding Y., Hang Q.L., Feng S.Q. (1999). Ga_2_O_3_ nanowires prepared by physical evaporation. Solid State Commun.

[27] Kuang Q., Lao C.S., Wang Z.L., Xie Z.X., Zheng L.S. (2007). High-sensitivity humidity sensor based on a single SnO_2_ nanowire. J. Am. Chem. Soc.

[28] Kim D.S., Shimpi P., Gao P.X. (2010). Isothermal gas flow separation of helical ZnS nanowires and nanobelts. Sci. Adv. Mater.

[29] Ma C., Moore D., Ding Y., Li Z.L., Wang J. (2004). Nanobelt and nanosaw structures of II-VI semiconductors. Int. J. Nanotechnol.

[30] Shimpi P., Yadav S., Ramprasad R., Gao P.X. (2011). Conversion of [0001] textured ZnO nanofilm into [01–10] directed nanowires driven by CO adsorption: *In situ* carbothermal synthesis and complementary first principles thermodynamics simulations. J. Phys. Chem. C.

[31] Shimpi P., Liao K.-T., Lin H.J., Gao P.X. (2012). Conversion of functional nanofilm into nanowires using combination of *in situ* carbothermal and stress induced recrystallization. Sci. Adv. Mater.

[32] Gao P.X., Wang Z.L. (2002). Self-assembled nanowire-nanoribbon junction arrays of ZnO. J. Phys. Chem. B.

[33] Gao P.X., Wang Z.L. (2004). Nanopropeller arrays of zinc oxide. Appl. Phys. Lett.

[34] Ren Z., Guo Y.B., Wrobel G., Knecht D., Gao H.Y., Zhang Z.H., Gao P.X. (2012). Three dimensional koosh ball nanoarchitecture with tunable magnetic core, fluorescent nanowire shell and enhanced photocatalytic property. J. Mater. Chem.

[35] Shimpi P., Liao K.T., Xiao W., Gao P.X. (2012). Tunable photoluminescence in solution-processed ZnMgO nanowires through post-annealing atmosphere and substrate control.

[36] Park K.S., Lee J.-S., Sung M.-Y., Kim S.S. (2002). Structural and optical properties of ZnO nanowires synthesized from ball-milled ZnO powders. Jpn. J. Appl. Phys.

[37] Kling R., Kirchner C., Gruber T., Reuss F., Waag A. (2004). Analysis of ZnO and ZnMgO nanopillars grown by self-organization. Nanotechnology.

[38] Zhu L., Zhi M., Ye Z., Zhao B (2006). Catalyst-free two-step growth of quasialigned ZnMgO nanorods and their properties. Appl. Phys. Lett.

[39] Wang G., Ye Z., He H., Tang H., Li J. (2007). Growth and properties of ZnO/hexagonal ZnMgO/cubic ZnMgO nanopagoda heterostructures. J. Phys. D Appl. Phys.

[40] Hsu H.C., Wu C.Y., Cheng H.M., Hsieh W.F. (2006). Band gap engineering and stimulated emission of ZnMgO nanowires. Appl. Phys. Lett.

[41] Ohtaomo A., Kawasaki M., Koida T., Masubuchi K., Koinuma H., Sakurai Y., Yoshida Y., Yasuda T., Segawa Y. (1998). Mg*_x_*Zn_1-_*_x_*O as a II-VI widegap semiconductor alloy. Appl. Phys. Lett.

[42] Lin J.M., Cheng C.L., Lin H.Y., Chen Y.F. (2006). Giant enhancement of band gap emission in ZnO and SnO nanocomposites. Opt. Lett.

[43] Fu X.Q., Wang C., Feng P., Wang T.H. (2007). Anomalous photoconductivity of CeO_2_ nanowires in air. Appl. Phys. Lett.

[44] Chen Z., Lu C. (2005). Humidity sensors: A review of materials and mechanisms. Sens. Lett.

[45] He M., Qiu J., Liang X., Lu H.B., Jin K.J. (2007). Thickness-dependent surface morphology of La_0.9_Sr_0.1_MnO_3_ ultrathin films. Appl. Surf. Sci.

[46] Liao J.H., Lo Y.S., Wu T.B. (2008). Surface characterization of ultrathin La_0.75_Sr_0.25_MnO_3_ epitaxial films on SrTiO_3_ substrate. J. Cryst. Growth.

[47] Rostamnejadi A., Salamati H., Kameli P., Ahmadvand H. (2009). Superparamagnetic behavior of La_0.67_Sr_0.33_MnO_3_ nanoparticles prepared via sol–gel method. J. Magn. Magn. Mater.

[48] Wan Q., Li Q.H., Chen Y.J., Wang T.H., He X.L., Li J.P., Lin C.L. (2004). Fabrication and ethanol sensing characteristics of ZnO nanowire gas sensors. Appl. Phys. Lett.

[49] Feng P., Wan Q., Wang T.H. (2005). Contact-controlled sensing properties of flowerlike ZnO nanostructure. Appl. Phys. Lett.

[50] Chen Y.J., Xue X.Y., Wang Y.G., Wang T.H. (2005). Synthesis and ethanol sensing characteristics of single crystalline SnO_2_ nanorods. Appl. Phys. Lett.

[51] Singh V.N., Mehta B.R., Joshi R.K., Kruis F.E., Shivaprasad S.M. (2007). Enhanced gas sensing properties of In_2_O_3_:Ag composite nanoparticle layers; electronic interaction, size and surface induced effects. Sens. Actuators B Chem.

[52] Wang H., Huang H., Wang B. (2009). First-principles study of structural, electronic, and optical properties of ZnSnO_3_. Sol. State Commun.

[53] Inaguma Y., Yoshida M., Katsumata T. (2008). A polar oxide ZnSnO3 with a LiNbO_3_-Type structure. J. Am. Chem. Soc.

[54] Gou H., Zhang J., Li Z., Wang G., Gao F., Ewing R.C., Lian J (2011). Energetic stability, structural transition, and thermodynamic properties of ZnSnO_3_. Appl. Phys. Lett.

[55] Nasibulin A.G., Richard O., Kauppinen E.I., Brown D.P., Jokiniemi J.K., Altman I.S. (2002). Nanoparticle synthesis by copper (II) acetylacetonate vapor decomposition in the presence of oxygen. Aerosol Sci. Technol.

[56] Xu C.H., Woo C.H., Shi S.Q. (2004). The effects of oxidative environments on the synthesis of CuO nanowires on Cu substrates. Superlattices Microstruct.

